# The complete mitochondrial genome of an endangered mangrove plant: *Scyphiphora hydrophyllacea*

**DOI:** 10.1080/23802359.2020.1788460

**Published:** 2020-07-11

**Authors:** Yuechao Chen, Xianya Wei, Guangxuan Lin, Yiming Liu, Ying Zhang

**Affiliations:** a Guangdong Zhanjiang Mangrove National Nature Reserve Administration, Zhan Jiang, China; b Chengdu Agricultural College, Chengdu, China; c Life Science and Technology School, Lingnan Normal University, Zhanjiang, China

**Keywords:** *Scyphiphora hydrophyllacea*, Rubiaceae, mitochondrial genome, phylogenomic tree

## Abstract

The complete mitochondrial genome of an endangered mangrove plant: *Scyphiphora hydrophyllacea* was analyzed in this paper, which is the first for the genus within the family Rubiaceae. The mitogenome sequence is 354,155 bp in length containing 3 ribosomal RNA genes, 16 transfer RNA genes, and 37 protein-coding genes. Gene *ccmFc*, *ccmFn*, *rps3*, *rps13*, *rps10*, *rpl12*, *nad3* and *cox1* contain one intron, gene *cox2* and *atp9* contain three introns and gene nad1, *nad4* and *nad7* contain four introns. Furthermore, Gene *nad2* and *nad5* have five introns. Gene *nad1*, *nad2*, *nad5*, *nad7*and *Cox2* are trans-splicing genes. Phylogenetic analysis using the maximum likelihood method positioned *S. hydrophyllacea* closely with *Asclepias syriaca* in Gentianales.

*Scyphiphora hydrophyllacea* is one of the shrub mangrove plants belonging to Scyphiphora genus in the family Rubiaceae, which is a monotypic genus with a range from south India and Ceylon, Indochina and Hainan in China, through the Malay Archipelago and Philippines to tropical Australia and New Caledonia, Northward to the Solomon Islands and Palau (Tomlinson 1986). According to the categories and criteria of the International Union for the Conservation of Nature (IUCN) Red List of Threatened Species, *S. hydrophyllacea* was classified into the Least Concern (LC) category with the 20% global loss (Polidoro et al. 2010). Mitochondrial genome-based phylogenetic analysis would improve our understanding of the evolutionary relationship of this plant under tidal habitat. In this study, we sequenced and analyzed the complete mitochondrial DNA sequence of *S. hydrophyllacea*. This is the first complete mitogenome within the family Rubiaceae or even in Gentianales.

Fresh leaves were collected from one individual of *S. hydrophyllacea* in Sanya (N18°13′21.09″, E109°36′59.73″), Hainan Province, China. The total genomic DNA was extracted from three mixed fresh leaves of *S. hydrophyllacea* by using the modified CTAB method (Doyle 1987) in the laboratory of Hainan Normal University. Further, the specimen was also stored in the herbarium of Hainan Normal University (BHM-001). Genome sequencing was performed on an Illumina Hiseq X Ten platform with paired-end reads of 150 bp. In total, 50.4 Gb short sequence data with Q20 was 96.21% was obtained. The remaining high-quality reads were used to assemble the mitogenome using NOVOPlasty (Dierckxsens et al. 2016), where *Asclepias syriaca* (GenBank accession NC_022796.1) used as the seed sequence. The genes annotation in the mitogenome was doing with SPAdes v.3.9.0 (Bankevich et al. 2012) and some genes were annotated manually. The accession number in Genbank is MT610041. Four mitogenome sequences in Rosales were aligned including *S. hydrophyllacea*, *Aegiceras corniculatum*, one mangrove plant in Myrsinaceae was used as the out-group species. Phylogenetic analysis using the maximum likelihood algorithm was conducted with Mega X (Kumar et al. 2018).

The mitogenome of *S. hydrophyllacea* is 354,155 bp in length with GC content of 44.44%, which contains 3 ribosomal RNA genes (*rrn5*, *rrn18* and *rrn26*), 16 transfer RNA genes, and 37 protein-coding genes. Gene *ccmFc*, *ccmFn*, *rps3*, *rps13*, *rps10*, *rpl12*, *nad3* and *cox1* contain one intron, gene *cox2* and *atp9* contain three introns and gene *nad1*, *nad4* and *nad7* contain four introns. Gene *nad2* and *nad5* have five introns. Gene *nad1*, *nad2*, *nad5*, *nad7* and *Cox2* are trans-splicing genes. Phylogenetic analysis with other four plant mitogenomes showed that *S. hydrophyllacea* is closest with *Asclepias syriaca* in Gentianales (Figure 1). The useful genomic resources for characterization of genetic diversity of *S. hydrophyllacea* by the mitogenome will help for the study of evolution mechanism.

**Figure 1.**
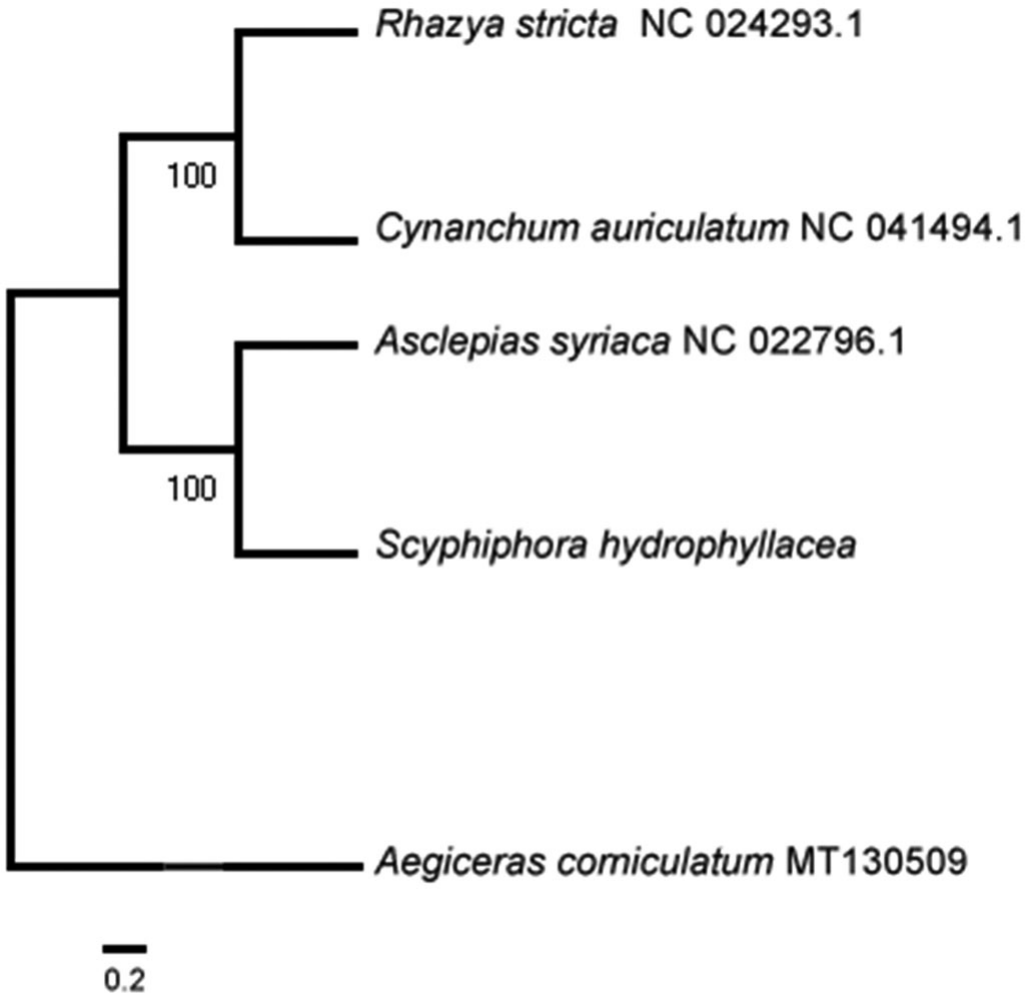
Maximum likelihood tree based on the sequences of five complete mitogenomes. Numbers in the nodes were bootstrap values from 1000 replicates. Scale in substitutions per site.

## Data Availability

The data that support the finding of this study are openly available in GenBank of NCBI at https://www.ncbi.nlm.nih.gov, reference number MT610041.

## References

[CIT0001] Bankevich A, Nurk S, Antipov D, Gurevich AA, Dvorkin M, Kulikov AS, Lesin VM, Nikolenko SI, Pham S, Prjibelski AD, et al. 2012. SPAdes: a new genome assembly algorithm and its applications to single-cell sequencing. J Comput Biol. 19(5):455–477. 22506599 10.1089/cmb.2012.0021 PMC3342519

[CIT0002] Dierckxsens N, Mardulyn P, Smits G. 2016. NOVOPlasty: de novo assembly of organelle genomes from whole genome data. Nucleic Acids Res. 45(4):e18. 10.1093/nar/gkw955 PMC5389512 28204566

[CIT0003] Doyle J. 1987. A rapid DNA isolation procedure for small quantities of fresh leaf tissue. Phytochem Bull. 19:11–15.

[CIT0004] Kumar S, Stecher G, Li M, Knyaz C, Tamura K. 2018. MEGA X: molecular evolutionary genetics analysis across computing platforms. Mol Biol Evol. 35 (6):1547–1549. 29722887 10.1093/molbev/msy096 PMC5967553

[CIT0005] Polidoro BA, Carpenter KE, Collins L, Duke NC, Ellison AM, Ellison JC, Farnsworth EJ, Fernando ES, Kathiresan K, Koedam NE, et al. 2010. The loss of species: mangrove extinction risk and geographic areas of global concern. PLOS One. 5(4):e10095. 20386710 10.1371/journal.pone.0010095 PMC2851656

[CIT0006] Tomlinson PB. 1986. The botany of mangroves. Cambridge: Cambridge University Press.

